# Ultrastructural examination of lung “cryobiopsies” from a series of fatal COVID-19 cases hardly revealed infected cells

**DOI:** 10.1007/s00428-022-03308-5

**Published:** 2022-03-16

**Authors:** Katia Cortese, Gudrun Holland, Lars Möller, Maria Cristina Gagliani, Emanuela Barisione, Lorenzo Ball, Paolo Pelosi, Federica Grillo, Luca Mastracci, Roberto Fiocca, Michael Laue

**Affiliations:** 1 Cellular Electron Microscopy Laboratory, Department of Experimental Medicine (DIMES), Human Anatomy, University of Genoa, Genoa, Italy; 2 National Consultant Laboratory for Electron Microscopy of Infectious Pathogens, Centre for Biological Threats and Special Pathogens 4 (ZBS 4), Robert Koch Institute, Seestr. 10, 13353 Berlin, Germany; 3 Interventional Pneumology Unit, Polyclinic San Martino University Hospital, Genoa, Italy; 4 IRCCS Policlinico San Martino University Hospital, Genoa, Italy; 5 Anesthesia and Intensive Care Unit, Department of Surgical Science and Integrated Diagnostics (DISC), University of Genoa, Genoa, Italy; 6 Anatomic Pathology Unit, Department of Surgical Sciences and Integrated Diagnostics (DISC), University of Genoa, Genoa, Italy

**Keywords:** SARS‑CoV‑2, Electron microscopy, Respiratory distress syndrome, Coronavirus, Virus particle, Diffuse alveolar damage

## Abstract

Ultrastructural analysis of autopsy samples from COVID-19 patients usually suffers from significant structural impairment possibly caused by the rather long latency between death of the patient and an appropriate sample fixation. To improve structural preservation of the tissue, we obtained samples from ventilated patients using a trans-bronchial “cryobiopsy” within 30 min after their death and fixed them immediately for electron microscopy. Samples of six COVID-19 patients with a documented histopathology were systematically investigated by thin section electron microscopy. The different samples and areas inspected revealed the ultrastructural correlates of the different phases of diffuse alveolar damage, including detachment of the alveolar epithelium, hyperplasia of type 2 cells, exudates, and accumulation of extracellular material, such as the hyaline membranes and fibrin. Macrophages and neutrophilic granulocytes were regularly detected. Structural integrity of endothelium was intact in regions where the alveolar epithelium was already detached. Aggregates of erythrocytes, leukocytes with fibrin, and thrombocytes were not observed. Coronavirus particles were only found in and around very few cells in one of the six patient samples. The type and origin of these cells could not be assessed although the overall structural preservation of the samples allowed the identification of pulmonary cell types. Hence, the observed alveolar damage is not associated with virus presence or structural impairment due to ongoing replication at later stages of the disease in fatal cases, which implies that the lung damage in these patients is at least propagated by alternative mechanisms, perhaps, an inappropriate immune or stress response.

**Supplementary Information:**

The online version contains supplementary material available at 10.1007/s00428-022-03308-5.

## Introduction

Since the emergence of COVID-19 in late 2019, caused by the severe acute respiratory syndrome coronavirus 2 (SARS-CoV-2) [1], tremendous efforts have been made to understand and to treat this disease [2]. And although approved vaccines reduce virus transmission and a severe outcome of the disease [3], the global pandemic continues to progress, resulting in significant loss of life and the appearance of new SARS-CoV-2 variants of concern. It is therefore still important to improve our understanding of the disease and to develop further preventative and therapeutic strategies.

SARS-CoV-2 infects the epithelium of the upper and lower airways and thereby causes COVID-19 disease [4]. Although the vast majority of COVID-19 patients are asymptomatic or mildly ill (approximately 80%), a still significant subset of patients shows a severe progression of the disease leading to pneumonia which is characterized by hypoxemia [5]. Critical cases (approx. 5%) develop an acute respiratory distress syndrome (ARDS), septic shock, or multiorgan failure [5]. Histopathology of lungs from autopsies has revealed different stages of diffuse alveolar damage (DAD) and severe capillary congestion, microthrombosis, and thrombosis of small to midsized arteries suggesting vascular dysfunction [6]. High quality ultrastructural correlates of the histopathological findings have been demonstrated only sporadically (e.g., by Ochs and colleagues [7]).

More recent histopathology and immunology data suggest that, in addition to direct viral effects, lung lesion in COVID-19 might be driven by the activation and/or an imbalance of the innate immune system [8, 9]. This hypothesis is supported by single cell analysis at the proteome and transcriptome level which demonstrated the association of an upregulation of specific components of the innate immune system in COVID-19 patient cells with pathological lung damage [10–12]. Accordingly, tissue-based detection of SARS-CoV-2 in multiple organs by immunohistochemistry (IHC) and in situ RNA hybridization (ISH) suggests that viral tissue damage may be a transient phenomenon that is generally not sustained throughout disease progression [13]. However, detection of SARS-CoV-2 by IHC suffers from a low specificity and both, IHC and ISH, only show the presence of protein or RNA but not necessarily of the intact virus, which may lead to misinterpretation of the pathogenesis [14].

Detection of SARS-CoV-2 particles in autopsy material by electron microscopy (EM) could provide direct evidence for the presence of the virus in the cellular context but seems to be challenging because it has led to many misinterpretations, presumably because of the poor preservation of autopsy samples and the difficulty to find and to recognize viral particles in such material [15–17]. To date, only few papers convincingly demonstrated presence of virus particles in the respiratory system at the ultrastructural level, but all data suffer from a limited structural preservation of the tissue and also a rather small sample size [15, 18, 19]. In summary, the localization of virus particles in patients lung tissue and its relation to the pathological changes of the tissue are largely unknown. Thus, the aim of the present study was to systematically investigate larger parts of well-preserved lung samples by electron microscopy for the ultrastructural correlates of the histopathological findings and for the presence of coronavirus particles. For this purpose, we used lung “cryobiopsies” from a case study of pathologically well-characterized COVID-19 patients [20]. Trans-bronchial “cryobiopsies” were taken within 30 min after exitus of the patients and directly fixed for thin section EM. All samples showed sufficient ultrastructural preservation to identify relevant cell types morphologically, which made it possible to systematically analyze a total section area of at least 10 mm^2^ taken at regular intervals from a cubic millimeter of each “cryobiopsy” sample. Large-scale tile scans of representative sections were provided as public data sets for further analysis.

## Materials and methods

### Patient characteristics and trans-bronchial lung cryobiopsy

Eight patients have been included in a pathological case study on COVID-19 patients in Genoa (Italy) [20]. Samples from six of those patients (patients C03 to C08) were available for an extensive ultrastructural analysis presented here. The patient characteristics and histopathological diagnosis relevant for the study are listed in Suppl. Table 1. Samples were taken by using a trans-bronchial lung cryobiopsy technique within 30 min after exitus of the still ventilated patient [20]. To preserve the tissue for electron microscopy, frozen tissue was immediately immersed in buffered glutaraldehyde (2.5% in 0.1 M cacodylate buffer) and stored for at least 1 week at 4 °C. Control cryobiopsy samples were obtained from then unaffected area of a living patient with nonspecific interstitial pneumonia and fibrosis using the same procedures.

### Sample preparation for thin section electron microscopy

Samples were post-fixed in 1% osmium tetroxide and 1% aqueous uranyl acetate for 1 h, each, dehydrated by a graded series of ethanol and embedded in epoxy resin (Poly-Bed; Polysciences, Inc., Warrington, PA). Sections were produced using ultramicrotomes from Leica, either at 300 to 500 nm (semithin) or at 50 to 100 nm (ultrathin) thickness. Semithin sections were transferred on glass slides and stained with toluidine blue. Ultrathin sections were either collected on naked copper grids and contrasted with uranyl acetate and lead citrate or on small pieces of a silicon wafer and contrasted with lead citrate. A thin carbon coating stabilized the ultrathin sections on grids for the imaging by transmission electron microscopy.

### Sample investigation by light and electron microscopy

Semithin sections were photographed with an inverted light microscope (ApoTome2, Carl Zeiss Microscopy), using a color CMOS camera (AxioCam 503 color, Carl Zeiss Microscopy), a 40x/1.1 NA water immersion objective and automated image montaging provided by the camera software (ZEN blue, version 2.6). Transmission electron microscopy (TEM) of ultrathin sections was performed by a group of operators using the following microscopes: (1) HITACHI 7800 (Hitachi), equipped with a tungsten filament operated at 100 kV; (2) Tecnai Spirit (Thermo Fisher Scientific), equipped with a LaB_6_ filament operated at 120 kV; and (3) JSM-2100 (Jeol), equipped with a LaB_6_ filament operated at 200 kV. Digital images presented in the manuscript were either acquired with a CCD camera (MegaviewIII, EMSIS) or a CMOS camera (Phurona, EMSIS). Montages of images were produced by using the respective camera acquisition software (iTEM, version 5.2, OSIS; Radius, version 2.1, EMSIS).

Large montages (up to 200 images) were recorded from thin sections collected on silicon wafers using a scanning electron microscope (Teneo, Thermo Fisher) and an in-column detector (T1) at 2 kV, 25–100 µA, 2–3 mm working distance. Large-field scans or tiled scans were recorded with a resolution of 1.8 to 49 nm per pixel, a dwell time of 3 µs and a line integration of 2. Stitching of the recorded images was either performed with the MAPS software (version 2.5, Thermo Fisher) used for acquisition or with the TrakEM module of Fiji [21].

We investigated six “cryobiopsy” samples from six deceased patients at the ultrastructural level. After an initial inspection of sections from all samples, which showed that the ultrastructural preservation was sufficient for a detailed analysis, we employed a systematic investigation strategy. From each sample, we took sections (semithin and ultrathin) with at least 1 mm^2^ surface area and a total depth of 1 mm at intervals of 100 µm or less. At least one thin section per section level was investigated by an expert in diagnostic EM using TEM. Mean investigation time per section was at least 1 h. The detection of viruses followed morphological criteria which are unique for coronaviruses [22].

## Results

**Fig. 1:**
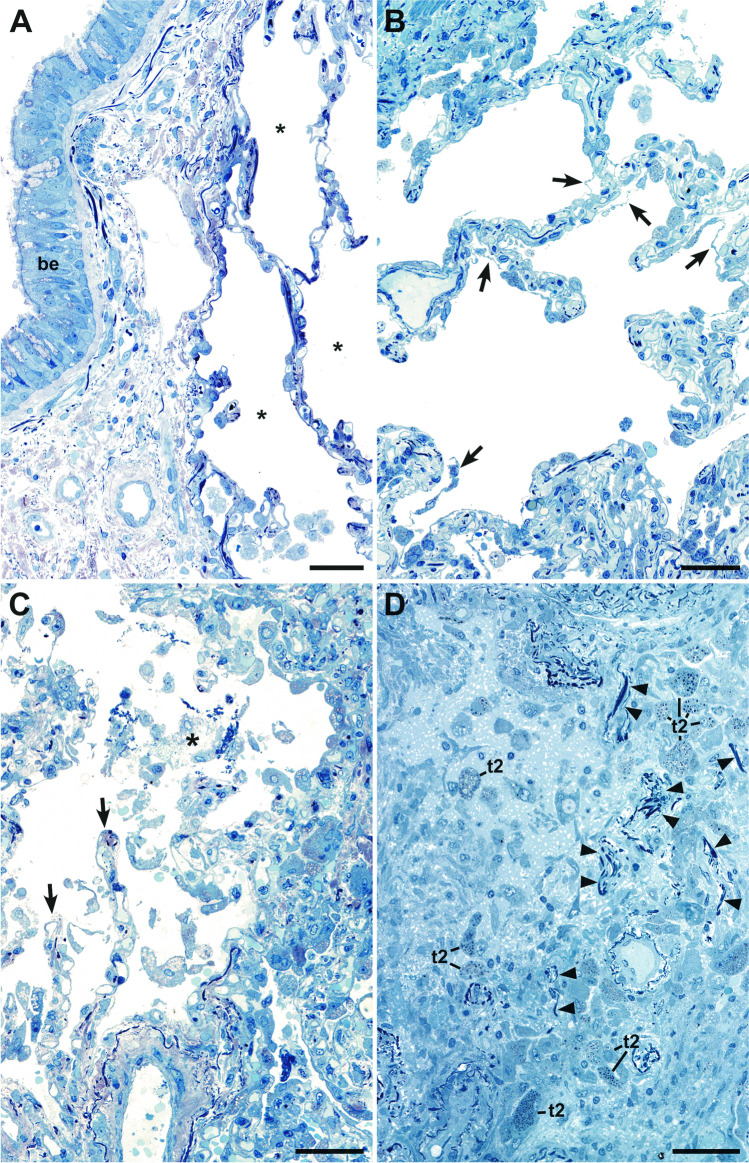
Representative semithin section areas demonstrate the variety of the alveolar histology of the samples analyzed. **A** Normal histology of alveolus (*) and of the bronchiolar epithelium (be) (patient C06). **B** Normal alveolar organization with locally detached alveolar epithelium (arrows) (patient C04).** C** Partially dissolved alveolar organization with few detectable alveolar septae (arrows) and an alveolar cavity, which is filled with detached cells and probably fibrin (*) (patient C07).** D** Completely dissolved alveolar organization (patient C05). Alveolar septae are not detectable. Elastic fibers, characterized by their dense appearance and filamentous morphology (arrowheads), may indicate remainders of alveolar septae. Many cells are embedded in an extracellular matrix. Some cells, frequently arranged in groups, contain conspicuous dark inclusions and represent type 2 cells (t2). Scale bar = 50 µm. Data sets 1–6

Light microscopy of semithin sections allowed the assessment of the general histology of the investigated sample areas (Fig. 1, Table 1). Large image montages of the first and last semithin section from all samples are available for digital microscopy (data sets 1–6). All samples contained alveoli, at least in some of the sections that were inspected. Significant impairment of alveolar architecture, i.e., congestion of the alveolar cavity and/or disintegration of alveolar septae, was found in samples of patients C03, C04, C05, and C07, with a restriction to some of the alveolar regions visible in patients C04 and C05 (Table 1). Alveolar architecture of samples from patients C06 and C08 appeared unaffected. However, even in samples with normal alveolar architecture, histopathological changes, such as focal detachment of the alveolar epithelium, over-representation of type 2 cells and of granulocytes could be detected (see below).

**Table 1:** General histological description of samples analyzed by electron microscopy

Patient	Comprehensive histologic DAD phase^1^	General histologic description of EM samples according to the inspection of semithin sections
CO3	Mid-phase	Impaired alveolus; small vessels
CO4	Late phase	Alveolus, partially impaired; bronchioles (epithelium and glands); small vessels
CO5	Late phase	Alveolus, partially impaired; small vessels
CO6	Early phase	Alveolus; bronchioles (epithelium and glands); cartilage; small vessels
CO7	Mid-phase	Impaired alveolus; small vessels
CO8	Early phase	Alveolus; small vessels; large vessel (second half of the inspected volume)

^1^From Barisione and colleagues[20]

**Fig. 2:**
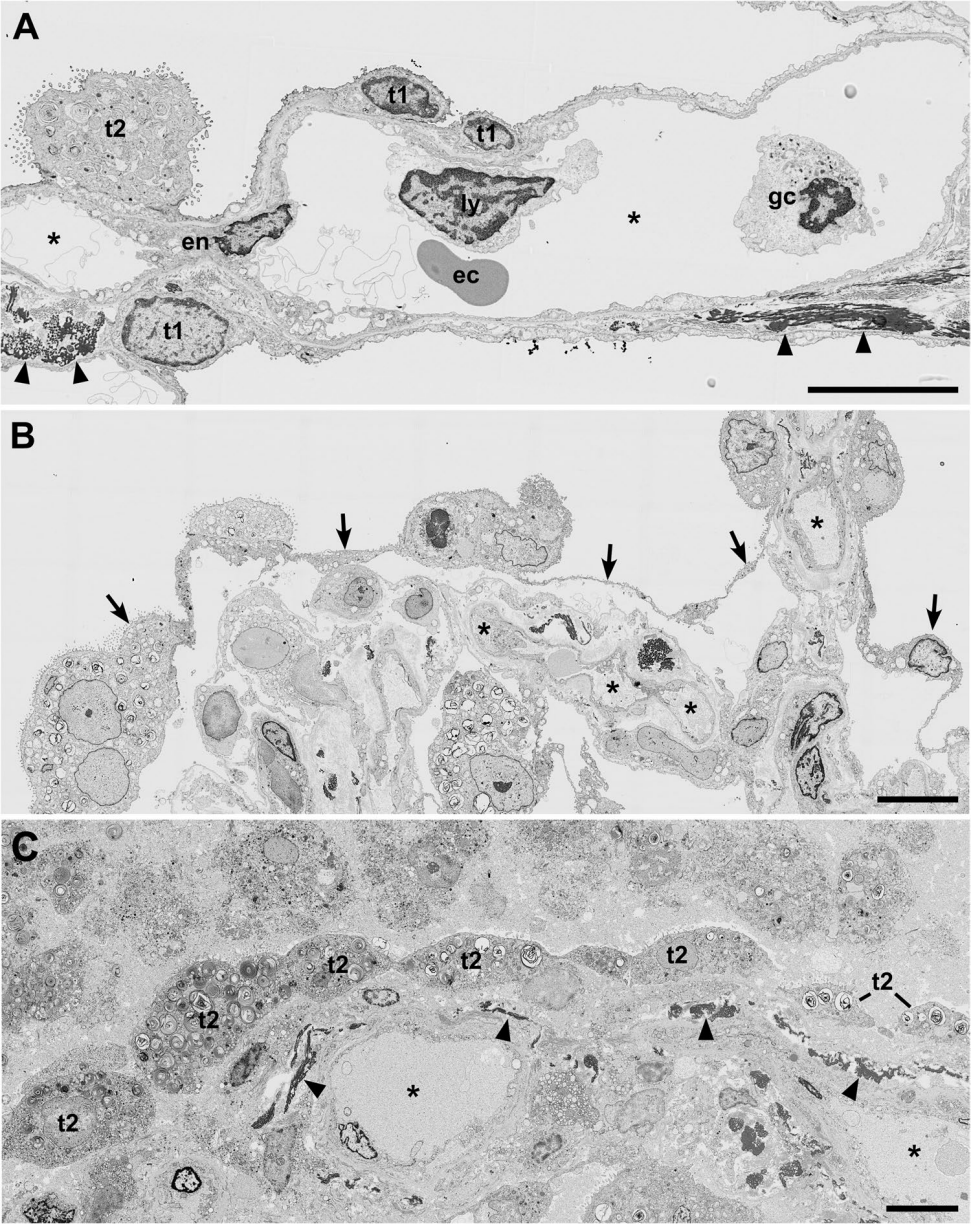
Alveolar septae with different degree of structural modification. **A** Intact alveolar septum with type 1 (t1) and type 2 (t2) cells and a capillary (*) formed by an endothelial cell (en) (patient C06). The lumen of the larger cavity of the capillary shows profiles of an erythrocyte (ec), a lymphocyte (ly), and a granulocyte (gc). Wider parts of the septae show elastic fibers (arrowheads). **B** Alveolar septum with detached alveolar epithelium (arrows) (patient C04). **C** Sample region with dissolved alveolar septum structure (patient C05). Larger capillaries (*) are visible and an epithelium-like layer of type 2 cells (t2) which separate the capillaries and connective tissue, such as elastic fibers (arrowheads), from an undefined cell mass above. Scale bar = 10 µm. Data set 7

**Fig. 3:**
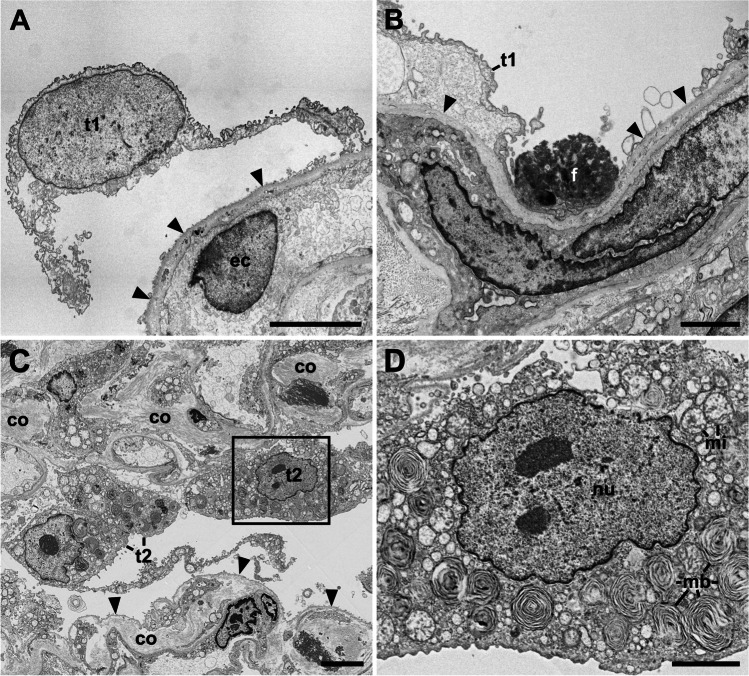
Pathological changes of the alveolar epithelium. **A**, **B** Type 1 cells (t1) detach from the basal membrane (arrowheads). ec, endothelial cell; f, fibrin (patient C08). **C**, **D** Type 2 cell (t2) aggregate (patient C04). **C** Partial overview of two alveolar septae which show a type 2 cell aggregate (upper septum) or a denuded basement membrane (arrowheads; lower septum). Note that the septal walls are widened by increased deposition of collagen (co) (see Suppl. Figure 4 for a magnified view of the collagen). **D** Magnification of the type 2 cell marked in **C** which show the nucleus (nu) and many multi-lamellar bodies (mb). mi, mitochondrion. Scale bar in **A**, **C** = 5 µm; **B**, **D** = 2 µm. Data set 8

**Fig. 4:**
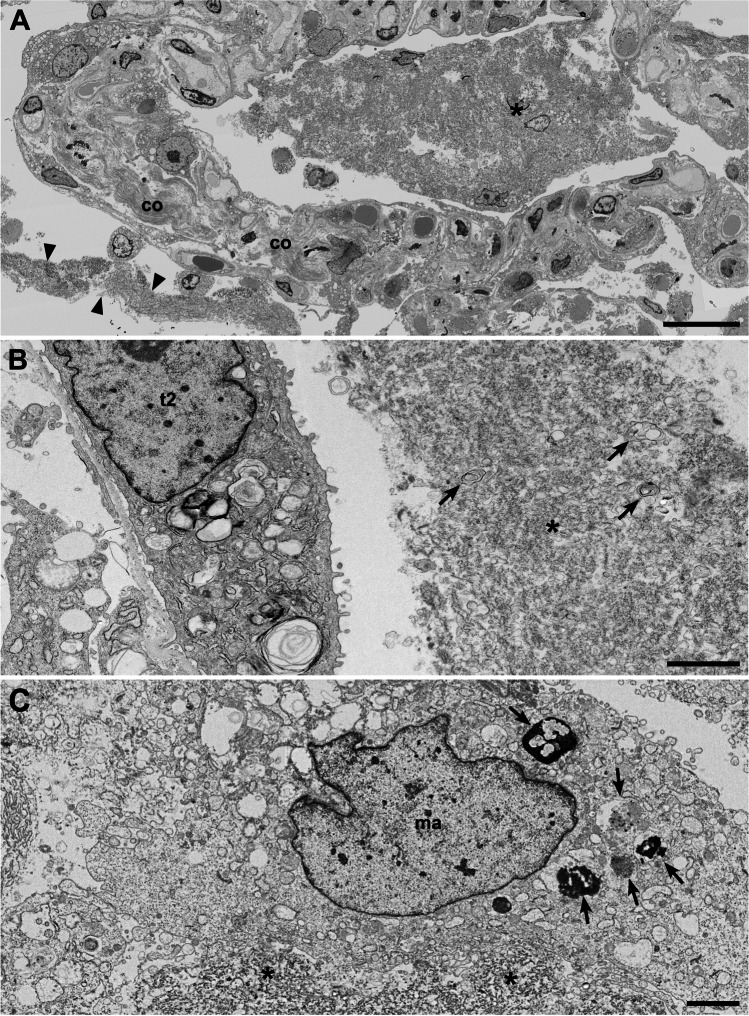
Accumulation of cells and debris in the alveolar cavity (patient C03). **A** Overview of a section through a structurally modified alveolus which shows an accumulation of rather amorphous material and some cells (*). In the lumen of an adjacent alveolus, a band-like accumulation of amorphous material is visible (arrowheads) which most probably is identical with the so-called hyaline membrane detected by histopathology. Increased deposition of collagen (co) is visible in the alveolar septum (see Suppl. Figure 4 for a magnified view of the collagen). **B** Higher magnification of a band-like material accumulation (hyaline membrane) in an alveolar space above a type 2 cell (t2) which shows a granular, but rather amorphous matrix (*) with some vesicular and lamellar structures (arrows) included. **C** A macrophage-like cell (ma) which is localized at the rim of accumulated extracellular material (*) and which contains phagolysosomes or lysosomes (arrows). Scale bar **A** = 20 µm; **B**, **C** = 2 µm. Data set 9

**Fig. 5:**
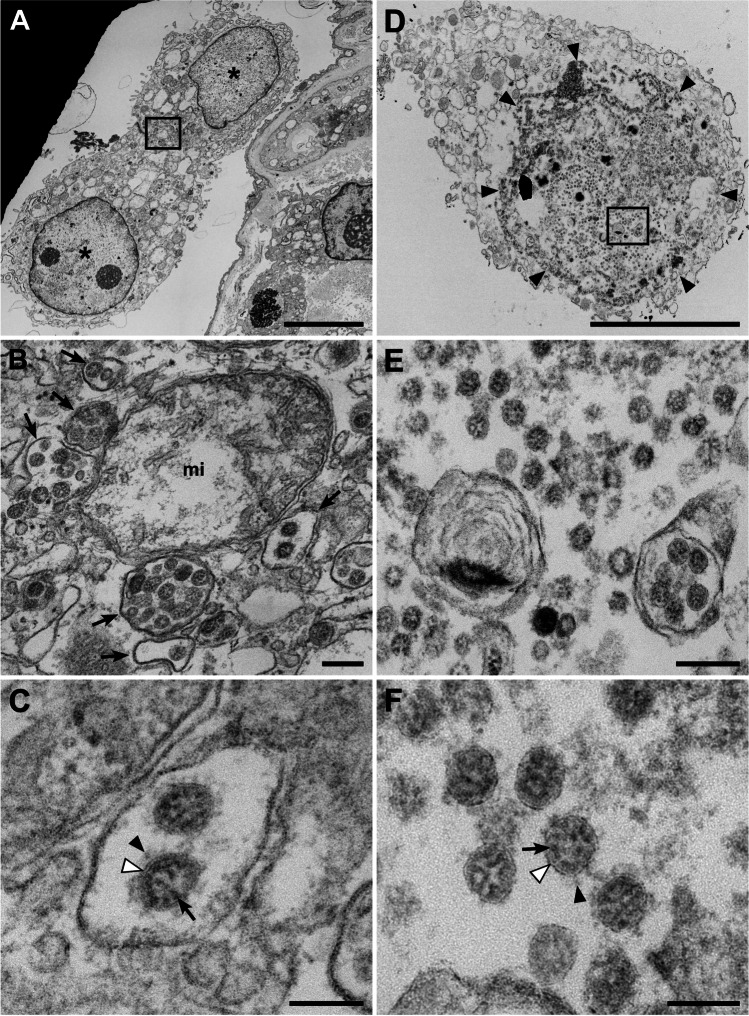
Virus-infected cells (patient C08). **A**–**C** Two isolated epithelial cells (*) of unknown identity which contain coronavirus particles in the cytoplasm. **B** Higher magnification of the cytoplasmic region marked in image **A** by a rectangle which shows groups of virus particles enclosed in several membrane-bound compartments (arrows). mi, mitochondrion. **C** Detail of one of those compartments including two virus particles which show the typical features of coronavirus particles: enveloping membrane (white arrowhead), surface spikes with a globular tip (black arrowhead), granular content representing ribonucleoprotein (arrow), profile diameter between ~ 60 and 200 nm [22]. **D**–**F** An isolated cell of unknown identity which reveals only few detectable organelles but many coronavirus particles in the cytoplasm. Note that the cytoplasmic region (marked by arrowheads) which contains the virus particles appears to be structurally separated from other parts of the cytoplasm without revealing a clearly detectable delimiting membrane. **E**, **F** Higher magnifications of the virus particles of the region marked in image **D** demonstrate the typical features of coronavirus particles (see description for image **C**). Scale bar **A**, **D** = 5 µm; **B**, **E** = 200 nm; **C**, **F** = 100 nm. Data set 10

Electron microscopy of thin sections revealed a sufficient structural preservation for ultrastructural analysis. Cellular integrity in most cells was documented by the presence of the plasma membrane and major cell organelles, such as nucleus, mitochondria, endoplasmic reticulum, Golgi apparatus, and vesicles (see Figs. 2, 3, 4, and 5, Suppl. Figures 1–7, and data sets 7–18). However, a few organelles generally showed a striking ultrastructure in the samples. The lumen of the rough endoplasmic reticulum (rER) often revealed circular profiles which derived from invaginations of the rER membrane (Suppl. Figure 1) and which could be confused with coronavirus particles at first sight. However, the internal granules of the profiles are identical in size to the ribosomes present at the outer rER membrane. Moreover, the distribution of most of the granules within the profiles revealed a clear association with the membrane, which also accounts for their identification as ribosomes (Bullock and colleagues reported similar structures with the same diagnosis [23]). A biopsy from a non-COVID-19 patient also showed such profiles within the rER (not shown), but at a very low frequency. Apart from the peculiar presences of profiles in the rER, the matrix of most mitochondria appeared extracted and their internal membranes were often largely missing (Figs. 3D, 5B, Suppl. Figure 2A). Inspection of the sample from the control patient also showed impairment of the matrix ultrastructure of mitochondria (Suppl. Figure 2B), but usually to a lesser degree and at a smaller fraction than the mitochondria in the samples from COVID-19 patients.

The appearance of the alveolar architecture correlated gradually with the presence and appearance of the alveolar epithelium. In virtually unaffected alveoli, the alveolar epithelium was formed by flat type 1 cells, which covered most of the alveolar septum surface, and more voluminous, but locally confined type 2 cells (Fig. 2A). Cellular detachment from the basal membrane, cell, and debris accumulation in such areas were usually not visible. Impairment of alveolar architecture, such as an irregular epithelial lining or widening of alveolar septae by increased deposition of collagen (Figs. 3C, 4A), which reduces alveolar space, was associated with a detachment of alveolar epithelial cells from the basal membrane (Figs. 2B, 3A). Alveoli affected by epithelia cell detachment often showed an increase in type 2 cells compared to unaffected alveoli (Fig. 2A, B), which indicates type 2 cell hyperplasia. In sample regions where the alveolar architecture was not or hardly detectable, type 2 cell clusters were regularly present (Figs. 2C, 3C, D, Suppl. Figure 3), and, besides elastic fibers, these were the only characteristic structural elements of the alveolar septum which could be identified.

Sample regions which showed modified alveolar septae or a more severe impairment of the alveolar architecture revealed the presence of accumulated extracellular material around the cells or in the remaining alveolar space (Fig. 4A). If septae were present, the material occasionally appeared as a continuous layer on top of the septum with its remaining constituents (Fig. 4B), which is likely identical with the hyaline membranes described by histopathology [24]. The extracellular material was of heterogenous composition, mostly granular with vesicles, membrane lamellae, or fragments of cells (Fig. 4). Larger accumulations of extracellular material were usually infiltrated by intact cells, which show structural signatures of macrophages (Fig. 4C) or, occasionally, of neutrophils (not shown). In sample regions with a completely dissolved alveolar architecture, cells were surrounded by the extracellular material which contained more cell fragments than the material in sample regions revealing an alveolar architecture (Suppl. Figure 3; see also original large-scale data set of the figure). Fibrin could be occasionally detected in regions where epithelial detachment from basal membrane was visible (Fig. 3B). In regions with more significant alveolar architecture impairment, fibrin accumulations could be detected regularly (Suppl. Figure 5A). Usually, cells with structural characteristics of macrophages or neutrophils were associated with such fibrin accumulations (Suppl. Figure 5).

Capillaries and blood vessels appeared rather inconspicuous. In regions of intact alveolar architecture and moderate degree of alveolar epithelial detachment, endothelial ultrastructure and capillary integrity were virtually unaffected (Suppl. Figure 6A). In more structurally impaired alveolar regions, the endothelial cells revealed vacuolization (Suppl. Figure 6B) and other structural signatures of cellular degeneration, such as plasma extraction and detachment from basement membrane. In contrast to the endothelial preservation, erythrocytes with the typical shape and density were only occasionally detected (e.g., Fig. 2A). In most capillaries, profiles of membrane-bound condensed sacs with a low luminal density were present (Suppl. Figure 6) which represent most probably erythrocyte ghosts. In the sample of a non-COVID-19 patient, many erythrocytes revealed a loss of matrix density which seemed to be extruded into the lumen of the capillary and, occasionally, similar membrane sacs as observed in the biopsies of the COVID-19 patients (not shown). Granulocytes, most probably mainly neutrophils, were regularly seen in capillaries of all samples from COVID-19 patients (Fig. 2A). Lymphocytes (Fig. 2A) and thrombocytes could be detected at much lower frequency within capillaries and blood vessels. A thrombus with fibrin and thrombocyte accumulation could not be detected. Fibrin was only exceptionally seen in capillaries and blood vessels.

Despite a careful examination of more than one hundred ultrathin sections by EM, we only found five infected cells in one patient sample (patient C08) with intracellular and a few extracellular particles that can be confidently assigned as coronavirus (Fig. 5). All of the infected cells had no obvious contact with an epithelium and their cell type could not be determined. Some of them seem to be detached from an epithelium, because they reveal cell–cell contacts (Fig. 5A). In comparison to other cells in the samples, especially the epithelial cells, the ultrastructural preservation appeared impaired, because the cytoplasm was extracted and organelles were only partially preserved. However, the cells showed many virus particles which reveal the typical size and features of coronaviruses [22], i.e., a size between 60 and 200 nm, a limiting biomembrane, spikes, and typical ribonucleoprotein profiles of the correct size (Fig. 5C, F). In most of the cells, the particles were localized in larger vesicles (Fig. 5A, B), but in structurally more impaired cells, virus particles were the only structures which could be clearly identified (Fig. 5D, E). Structural signatures of virus replication and assembly, such as double-membrane vesicles or buddings into the replication organelles, could not be detected in any of the cells inspected. It is noteworthy that the inspected samples also contained significant amount of bronchiolar tissue (Fig. 1A; Suppl. Figure 7), which was also negative for coronavirus particles and structural signatures of virus replication.

## Discussion

The structural preservation of “cryobiopsies” was sufficient to detect cell types of lung alveoli and even coronavirus particles, which is difficult in autopsy material, most probably because of the long intervals between time of death of the patient and conservation of the material for EM [15–17]. Remarkable structural peculiarities were round profiles within the rER, which were obviously produced by invagination from the cytoplasmic side, and a damage of the mitochondrial matrix structure. In a control biopsy from a non-COVID-19 patient, the profiles in the rER were rarely found, which suggests that their formation could be due to either the COVID-19 disease or to the fact that the control patient was alive during biopsy. In contrast, the mitochondrial damage was regularly present in the cryobiopsy of the control patient, albeit not in all mitochondria and at the same degree, which indicates that this damage could be induced by the freezing of the sample during the cryobiopsy procedure. Freezing and thawing of the tissue possibly caused osmotic stress, which could also be the reason for the poor preservation of most of the erythrocytes.

The histopathological aspects of the alveolar damage described for the six patient biopsies in this study are in line with the results already published for the patients by Barisione and colleagues [20]. The main observation was DAD, to various severity, with an accumulation of material and exudate in the remaining extracellular space, an enrichment of macrophages, neutrophilic granulocytes, and collagen. These results coincide with data from many other studies and reveal a considerable similarity with histopathological data from other cases showing ARDS [6, 8, 25]. Few and still limited ultrastructural investigations on the ultrastructural changes of the lung in COVID-19 patients have been published so far (e.g., [7, 26]). These studies underline the similarity between lung damage induced by COVID-19 and other cases of ARDS. The data of our more extended analysis reveal a striking similarity with detailed ultrastructural data presented for ARDS in other diseases [24, 27]. Alveolar damage seems to start with the detachment of alveolar epithelial cells. The endothelial cells appear intact in regions where the alveolar epithelial cells have started to detach and reveal intracellular changes, such as vacuolization, in regions of more severe alveolar epithelium damage. The structural integrity of the capillary endothelium at early stages of ARDS was also described for other cases of ARDS [24, 27] and in animal models [28] which suggests that the endothelial damage follows the damage of the alveolar epithelium and is not the cause for the latter. However, we cannot rule out the presence of molecular changes, e.g., in the cell contacts providing the barrier function of the endothelium, which were not directly visible at the ultrastructural level.

Virus particles were only detected in five cells from one patient (C08), which was also one of the two patients that were positive in IHC [20]. The cells revealed no typical internal features and were found isolated from any tissue which did not allow their identification. Two of them revealed cell–cell contacts which indicate an epithelial origin. Since virus detection was generally possible, we assume that we would have detected virus particles in other regions of the samples if they had been present. We also did not detect the typical double-membrane vesicles or convoluted membranes which are hallmarks of coronavirus replication in cell culture [17]. However, it is unclear if these hallmarks are also visible in infected patient cells. Afzelius showed a case of a coronavirus-infected, well-preserved trachea epithelium from a biopsy and did not report the presence of such compartments [29]. Most of the ultrastructural studies on COVID-19 patient material were performed on autopsy material and suffer from structural preservation which could have affected recognition of replication structures and virus particles [17]. In summary, except from the handful of unidentified cells, lung cells revealed no coronavirus particles and structural evidence of ongoing infection in the many sample areas we have inspected.

From the data we present here, we can conclude that the presence of virus particles and replication-induced structural modification of the cellular architecture is not necessary to propagate the alveolar damage observed, because we could not find virus particles or signatures of replication in or around the affected cells, not even in early stages of alveolar epithelium detachment or unaffected regions nearby. The infection, i.e., viral particles and structural modifications induced by viral replication, might have already been eliminated by intracellular processes, cell death, and/or digestion by macrophages. IHC results from the patients support this hypothesis because only patients at the early stage of DAD (patients C06 and C08, which was also positive by EM) showed immuno-positive alveolar cells with antibodies against the viral N-protein while patients at later stages of DAD were immuno-negative [20], which is in line with results from other studies [30]. The trend toward lower viral RNA copy number in later stages of the disease [8] or DAD [30] underpins this notion. Another explanation for the identification of few infected cells could be that we have missed rare or very focal infections with our still small sample size in comparison to the entire lung. However, it is generally questionable if a productive infection of many lung cells is possible, due to the low amount of entry receptors present [31], or even necessary to produce the lung damage observed. In ARDS, the host immune system initially is most probably hyper-activated by the various causes of ARDS, e.g., trauma or sepsis, which induces the release of danger- or pathogen-associated molecular patterns triggering the immune system and generating tissue damage [32]. In COVID-19, the activation of such an immune response could be initiated by a signal of a preceding or an ongoing infection transported from the upper respiratory system or from other regions of the lung, perhaps, from only focally infected groups of cells. Activated mobile cells, like monocyte-derived macrophages, or virus material, such as RNA, which are regularly found in the lung of COVID-19 patients [14, 33], could be initiators of the local (defense) response of the lung [34–36]. The results presented here suggest that progression of lung epithelial damage in fatal cases is independent of the presence and replication of the virus, supporting the hypothesis of a significant role of host response in the pathogenesis of lethal COVID-19.

## Supplementary Information

Below is the link to the electronic supplementary material.

Supplementary file1 (PDF 14190 KB)

## Data Availability

See Suppl. Tab. 2 for a detailed description of data sets available on the public repository zenodo. Further data or material is available from the corresponding author on request. Data set 01: https://doi.org/10.5281/zenodo.5681966 Data set 02: https://doi.org/10.5281/zenodo.5682151 Data set 03: https://doi.org/10.5281/zenodo.5682214 Data set 04: https://doi.org/10.5281/zenodo.5682238 Data set 05: https://doi.org/10.5281/zenodo.5682286 Data set 06: https://doi.org/10.5281/zenodo.5682578 Data set 07: https://doi.org/10.5281/zenodo.5682656 Data set 08: https://doi.org/10.5281/zenodo.5682693 Data set 09: https://doi.org/10.5281/zenodo.5682744 Data set 10: https://doi.org/10.5281/zenodo.5682815 Data set 11: https://doi.org/10.5281/zenodo.5682850 Data set 12: https://doi.org/10.5281/zenodo.5682881 Data set 13: https://doi.org/10.5281/zenodo.5682980 Data set 14: https://doi.org/10.5281/zenodo.5683073 Data set 15: https://doi.org/10.5281/zenodo.5683122 Data set 16: https://doi.org/10.5281/zenodo.5702444 Data set 17: https://doi.org/10.5281/zenodo.5702550 Data set 18: https://doi.org/10.5281/zenodo.5702609
