# Supplementary material for: Ultrastructural examination of lung “cryobiopsies” from a series of fatal COVID-19 cases hardly revealed infected cells
Source: Virchows Arch. 2022 Mar 16;480(5):967–77. doi: 10.1007/s00428-022-03308-5 (PMC8924574; doi:10.1007/s00428-022-03308-5)
Supplement: Supplementary file 1 — Supplementary file1 (PDF 14190 KB) [file 428_2022_3308_MOESM1_ESM.pdf]

Ultrastructural examination of lung ‚cryobiopsies' from a series of fatal COVID-19 cases hardly revealed infected cells

Katia Cortese[1], Gudrun Holland[2], Lars Möller[2], Maria Cristina Gagliani[1], Emanuela Barisione[3,5], Lorenzo Ball[4,5], Paolo Pelosi[4,5], Federica Grillo[5,6], Luca Mastracci[5,6], Roberto Fiocca[5], Michael Laue[2*]

[1] Cellular Electron Microscopy Lab, Department of Experimental Medicine (DIMES), Human Anatomy, University of Genoa, Genoa, Italy

[2] National Consultant Laboratory for Electron Microscopy of Infectious Pathogens, Centre for Biological Threats and Special Pathogens 4 (ZBS 4), Robert Koch Institute, Berlin, Germany

[3] Interventional Pneumology Unit, Polyclinic San Martino University Hospital, Genoa, Italy

[4] Anesthesia and Intensive Care Unit, Department of Surgical Science and Integrated Diagnostics (DISC), University of Genoa, Genoa, Italy

[5] IRCCS Policlinico San Martino University Hospital, Genoa, Italy

[6] Anatomic Pathology unit, Department of Surgical Sciences and Integrated Diagnostics (DISC), University of Genoa, Genoa, Italy

*Correspondence to:

Dr. Michael Laue

Robert Koch Institute

Seestr. 10

D-13353 Berlin

e-mail: lauem@rki.de

phone: ++49(0)30 18754 2675

Supplementary Material

Table of contents

Supplementary Tables..page 3

Reference...page 3

Supplementary Figure 1 ....page 4

Supplementary Figure 2 ....page 5

Supplementary Figure 3 ....page 6

Supplementary Figure 4 ....page 7

Supplementary Figure 5 ....page 8

Supplementary Figure 6 ....page 9

Supplementary Figure 7 ....page 10

Supplementary Table 1 Patient characteristics (taken from Barisione et al.)[1]

Patient	Gender (Male/ Female)	Age	Clinical cause of death	Days from onset to death	Days from hospitalization to death	Cryobiopsy for EM (site)
CO3	M	78	Cardiogenic shock	42	35	Left inferior lobe
CO4	M	59	Refractory hypoxaemia	31	30	Right inferior lobe
CO5	M	79	Refractory hypoxaemia	20	16	Right inferior lobe
CO6	F	77	Haemorragic shock	5	4	Left inferior lobe
CO7	M	69	Refractory hypoxaemia	38	33	Left inferior lobe
CO8	M	47	Cardiogenic shock	14	9	Left inferior lobe

Supplementary Table 2 Data sets

No.	Description	Microscope	Related to	Unique identifier
1	Complete first and last semithin section (patient C03)	LM	Fig. 1	DOI 10.5281/zenodo.5681966
2	Complete first and last semithin section (patient C04)	LM	Fig. 1	DOI 10.5281/zenodo.5682151
3	Complete first and last semithin section (patient C05)	LM	Fig. 1	DOI 10.5281/zenodo.5682214
4	Complete first and last semithin section (patient C06)	LM	Fig. 1	DOI 10.5281/zenodo.5682238
5	Complete first and last semithin section (patient C07)	LM	Fig. 1	DOI 10.5281/zenodo.5682286
6	Complete first and last semithin section (patient C08)	LM	Fig. 1	DOI 10.5281/zenodo.5682578
7	Alveolar modifications	EM	Fig. 2	DOI 10.5281/zenodo.5682656
8	Alveolar epithelial cells	EM	Fig. 3	DOI 10.5281/zenodo.5682693
9	Cells and debris in the alveolar cavity	EM	Fig. 4	DOI 10.5281/zenodo.5682744
10	SARS-CoV-2 particles in detached cells	EM	Fig. 5	DOI 10.5281/zenodo.5682815
11	Type-2-cell hyperplasia	EM	Suppl. Fig. 3	DOI 10.5281/zenodo.5682850
12	Endothelium	EM	Suppl. Fig. 5	DOI 10.5281/zenodo.5682881
13	Complete ultrathin section (patient C03)	EM	-	DOI 10.5281/zenodo.5682980
14	Complete ultrathin section (patient C04)	EM	-	DOI 10.5281/zenodo.5683073
15	Complete ultrathin section (patient C05)	EM	-	DOI 10.5281/zenodo.5683122
16	Complete ultrathin section (patient C06)	EM	-	DOI 10.5281/zenodo.5702444
17	Complete ultrathin section (patient C07)	EM	-	DOI 10.5281/zenodo.5702550
18	Complete ultrathin section (patient C08)	EM	-	DOI 10.5281/zenodo.5702609

Reference

1 Barisione E, Grillo F, Ball L, et al. Fibrotic progression and radiologic correlation in matched lung samples from COVID-19 post-mortems. Virchows Arch 2021; **478**: 471–85.

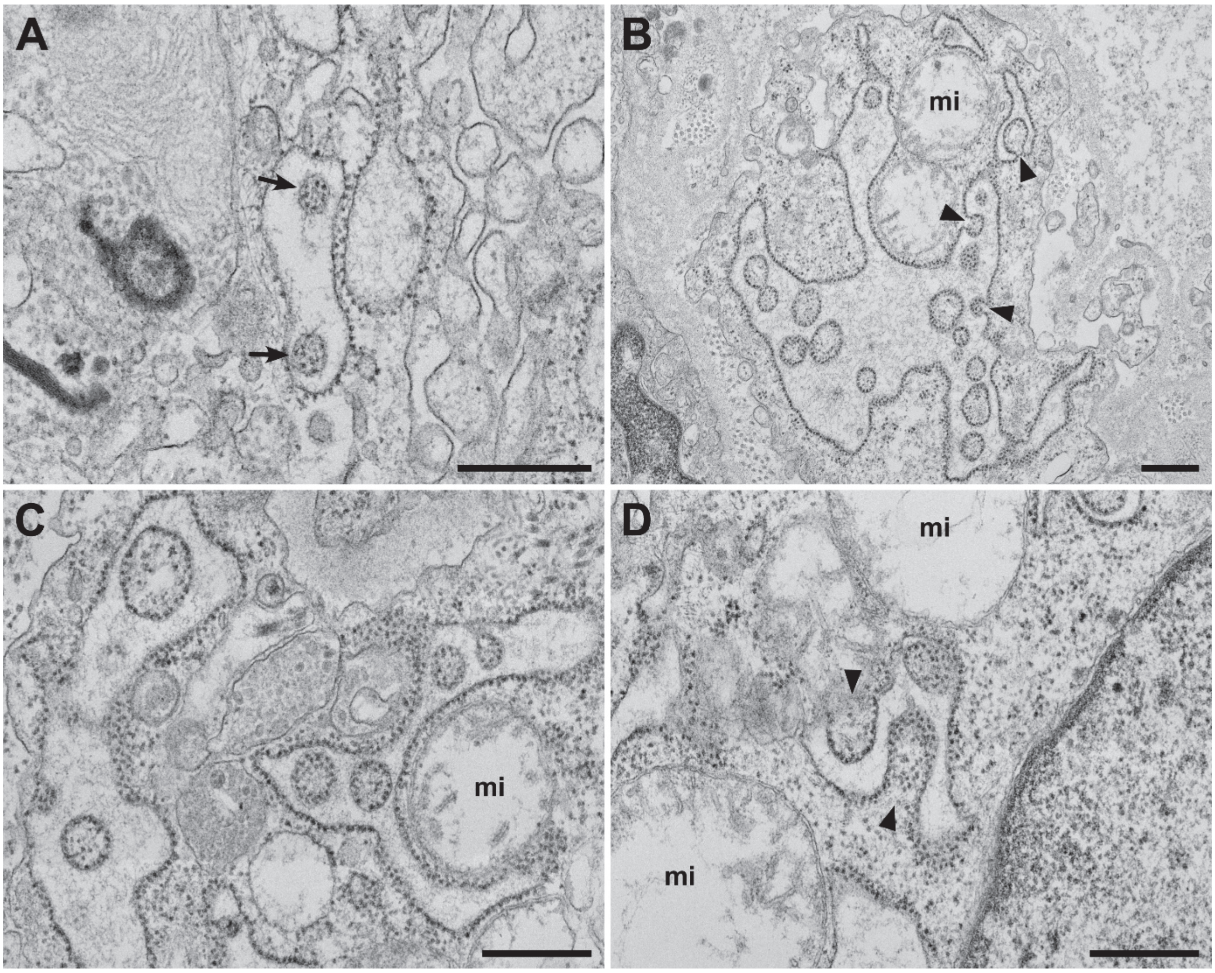

Suppl. Fig. 1 Vesicular profiles (*arrows*) within the cisternae of the rER in different cells (patient 08). Maximal diameter of the profiles varies between ~100 and 500 nm. The lumen of the profiles is filled with granules which resemble ribosomes regarding size, shape, density and regular decoration of the membrane. Invaginations from the cytoplasmic side of the rER into the lumen of the cisternae are visible in several regions (*arrowheads* in B, D). (*mi*) mitochondrion. Scale bars = 500 nm

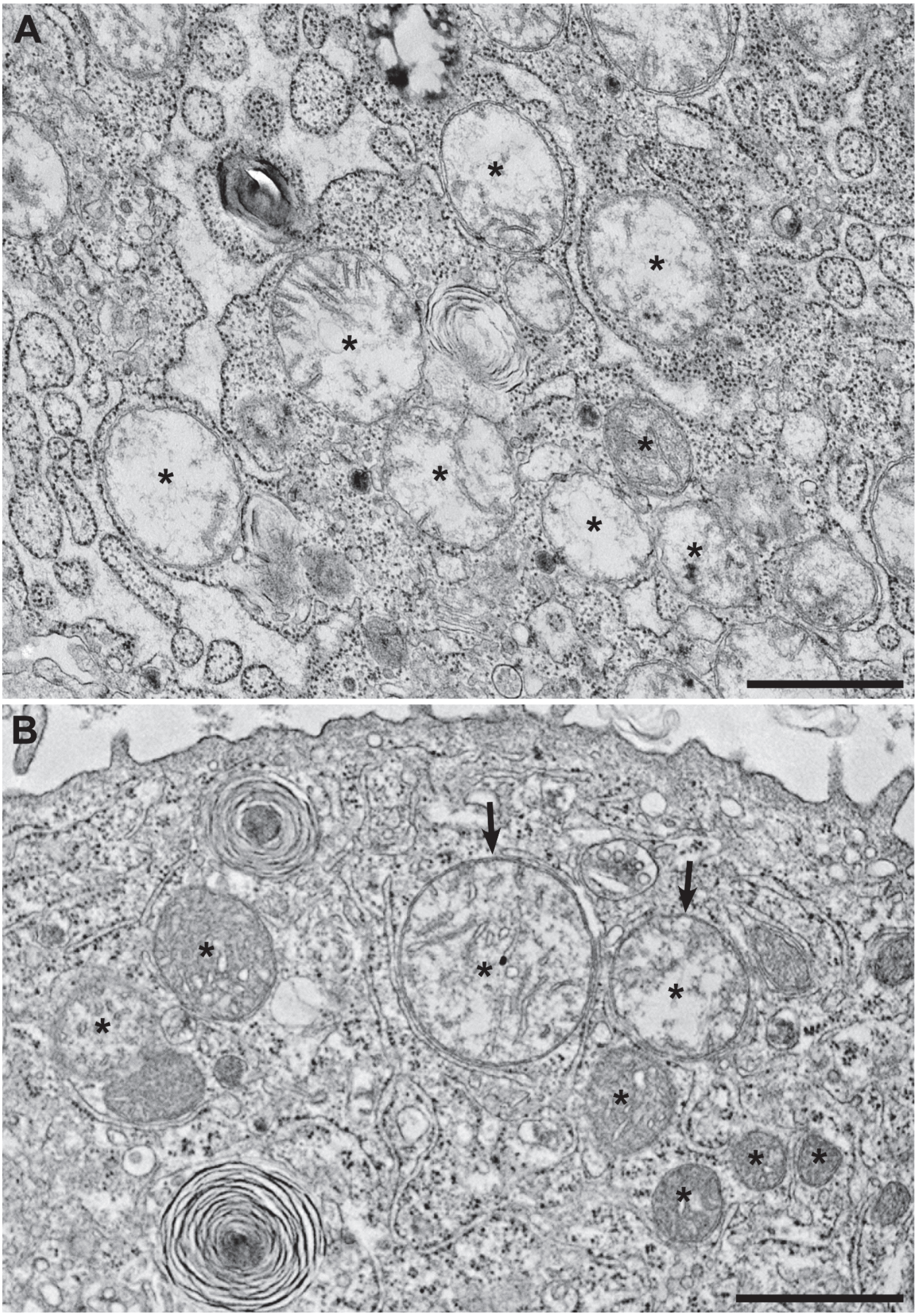

Suppl. Fig. 2 Ultrastructural appearance of mitochondria (*) in a sample from a COVID-19 patient (**A**; patient C08) and of a control patient (**B**). The control patient sample also shows mitochondria with a reduced matrix density (*arrows*) and dissolved inner membranes, which is the main appearance of mitochondria in samples from COVID-19 patients, but at a lower frequency than the sample from COVID-19 patients. Scale bars = 1 μm

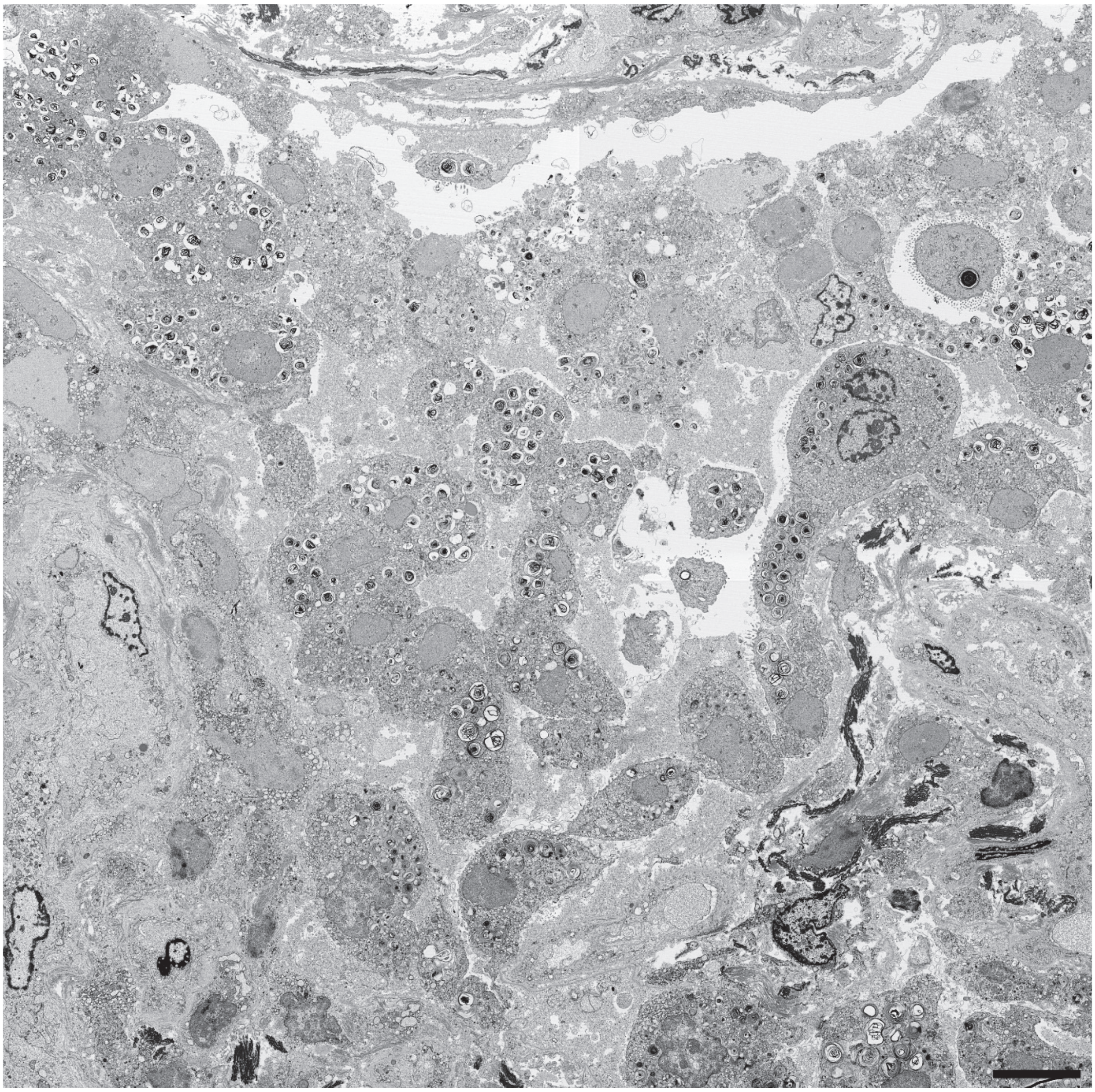

Suppl. Fig. 3 Massive type-2-cell hyperplasia in a sample area which reveals an entirely dissolved alveolar architecture. Type-2 cells are detectable by their prominent and numerous cytoplasmic multi-lamellar bodies which appear as dense-core, bright-lined inclusions. Patient C05. Scale bar = 10 µm. (data set 11)

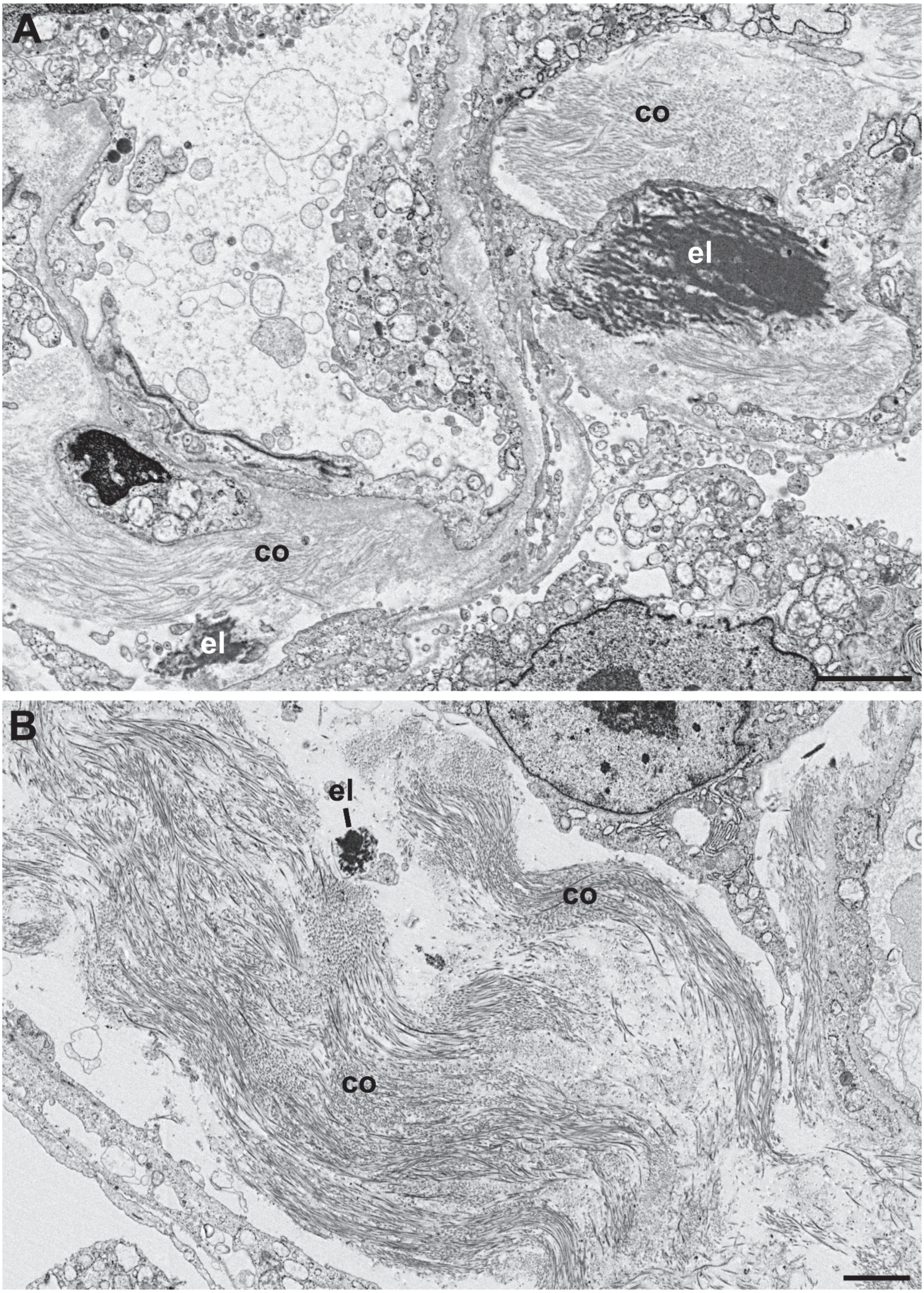

Suppl. Fig. 4 Collagen expansion in widened alveolar septae. Magnified view of collagen-rich regions indicated in Figure 3C (**A**) and Fig. 4A (**B**). Bundles of collagen fibrils (*co*) are visible in cross and in longitudinal section. Few aggregates of elastic fibres (*el*) are localized between the collagen bundles. Scale bar = 2 µm

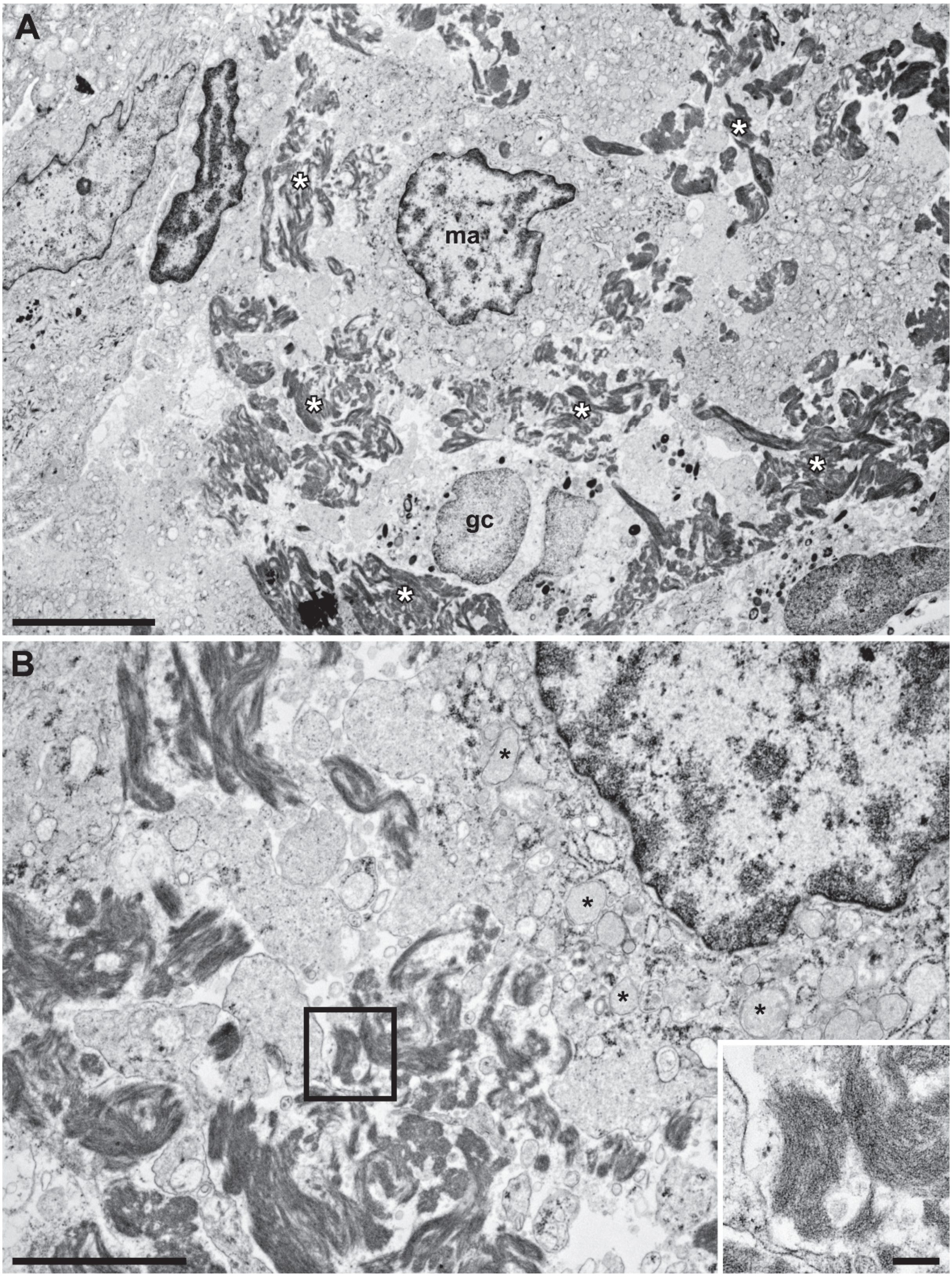

Suppl. Fig. 5 Fibrin accumulation in the alveolar space (patient C07). **A** Overview of the accumulated fibrin mass (*), which is infiltrated by a cell revealing structural signatures of a macrophage (*ma*) and another cell representing a granulocyte (*gc*). **B** Detail of the macrophage/fibrin interface. The cytoplasm of the macrophage-like cell reveals many large vesicles (*) which contain a fine-granular material. The region marked by the rectangle is enlarged in the inset and shows the characteristic fibrous substructure of the fibrin bundles. Scale bar in A = 5 µm, B = 2 µm, inset = 200 nm

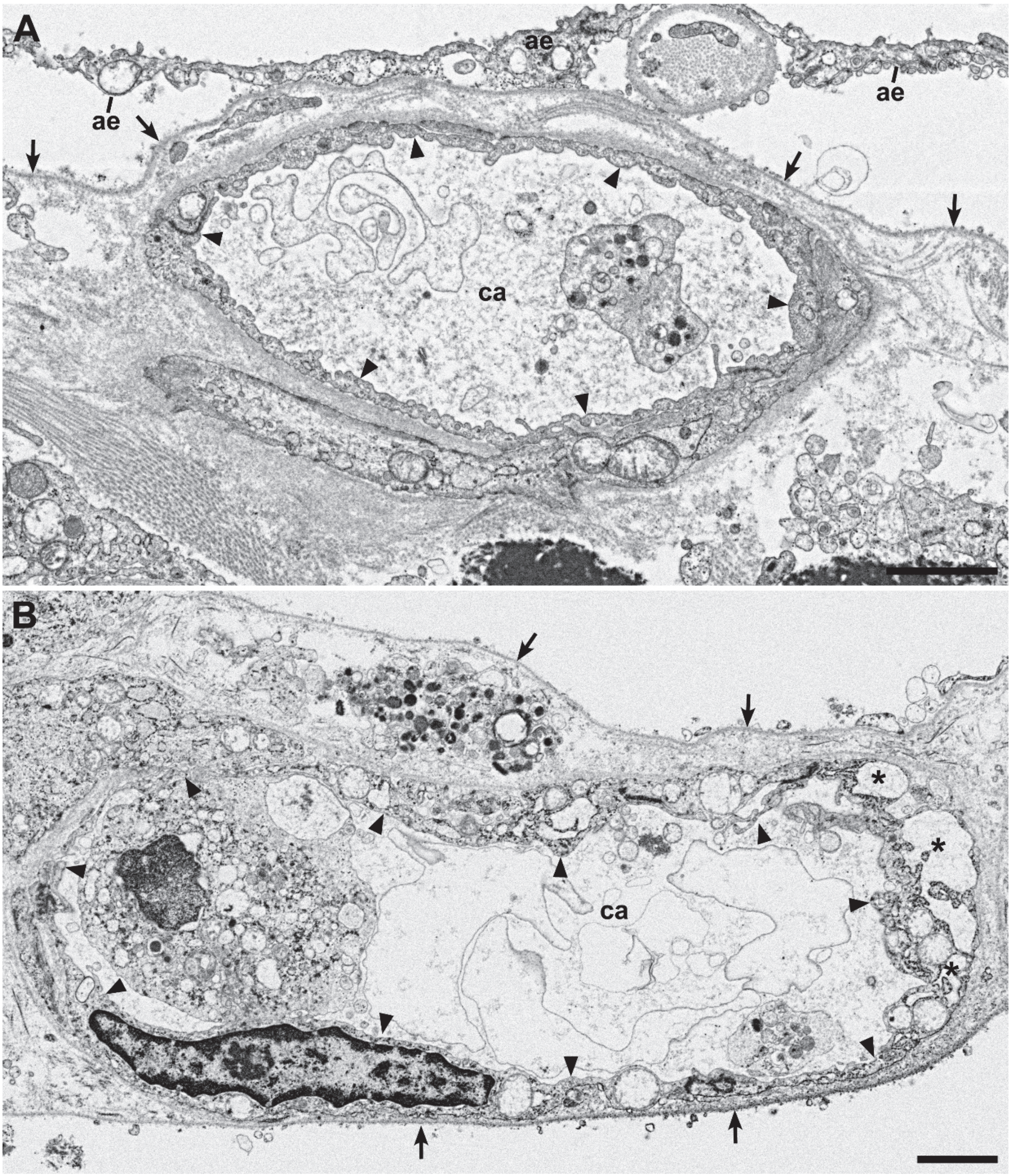

Suppl. Fig. 6 Integrity of the endothelium (*arrowheads*) in capillaries (*ca*). **A** Virtually intact endothelium in a sample region where the alveolar epithelium (*ae*) already has started to detach from the basal membrane (*arrows*). Patient C04. **B** Moderate endothelial damage in a sample region where the alveolar epithelium is completely absent (see the denuded basal membrane, *arrows*). The endothelium reveals regional vacuolization and thinning (*). Patient C07. Scale bar = 2 µm. (data set 12)

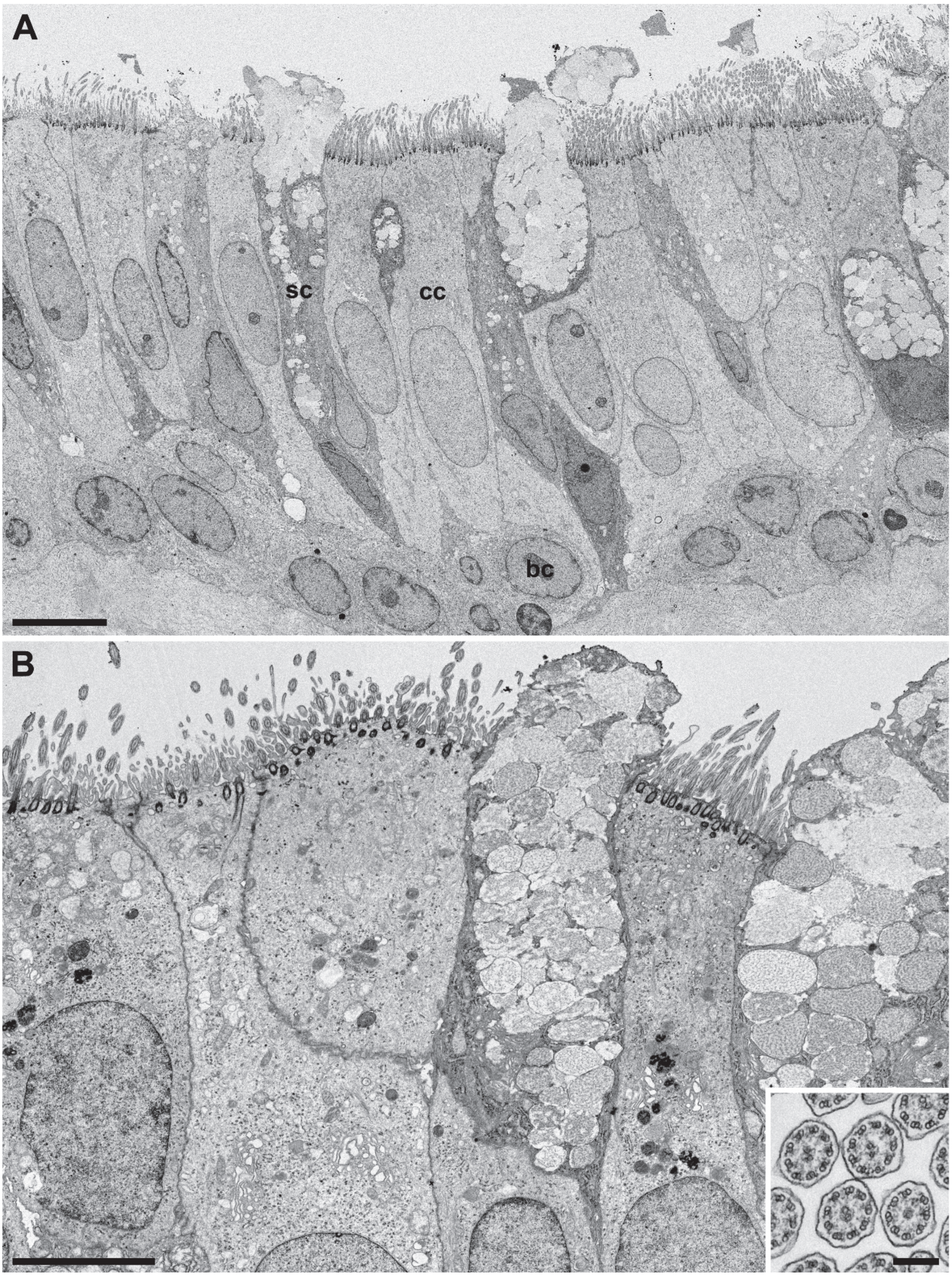

Suppl. Fig. 7 Bronchiolar epithelium (patient C06). **A** Overview of the intact epithelium formed by columnar ciliated (*cc*) and secretory cells (*sc*) and basal cells (*bc*). **B** Upper part of the columnar cells at higher magnification (different area than in A). Inset: Cross-section through the cilia reveal the typical interior microtubular organization. Scale bar in A = 10 µm, B = 5 µm, inset = 200 nm
